# Sustaining Transfers through Affordable Research Translation (START): study protocol to assess knowledge translation interventions in continuing care settings

**DOI:** 10.1186/1745-6215-14-355

**Published:** 2013-10-26

**Authors:** Susan E Slaughter, Carole A Estabrooks, C Allyson Jones, Adrian S Wagg, Misha Eliasziw

**Affiliations:** 1 Faculty of Nursing, Edmonton Clinic Health Academy, University of Alberta, Edmonton, AB T6G 1C9, Canada; 2 Faculty of Rehabilitation Medicine, Corbett Hall, University of Alberta, Alberta, AB T6G 2G4, Canada; 3 Faculty of Medicine and Dentistry, Campus Tower, University of Alberta, Alberta, AB T6G 1K8, Canada; 4 Faculty of Public Health and Community Medicine, Tufts University School of Medicine, Boston, MA 02111, USA

**Keywords:** Knowledge translation, Reminders, Continuing care, Health care aides, Long-term care, Older adults, Sustainability, Mobility

## Abstract

**Background:**

Bridging the research-practice gap is an important research focus in continuing care facilities, because the population of older adults (aged 65 years and over) requiring continuing care services is the fastest growing demographic among countries in the Organisation for Economic Co-operation and Development (OECD). Unlicensed practitioners, known as health care aides, provide the majority of care for residents living in continuing care facilities. However, little research examines how to sustain health care aide behavior change following initial adoption of current research evidence.

**Methods/Design:**

We will conduct a phase III, multicentre, cluster randomized controlled trial (RCT) using a stratified 2 × 2 additive factorial design, including an embedded process evaluation, in 24 supportive living facilities within the health zone of Edmonton, AB, Canada. We will determine which combination of frequency and intensity of reminders most effectively sustains the completion of the sit-to-stand activity by health care aides with residents. Frequency refers to how often a reminder is implemented; intensity refers to whether a reminder is social or paper-based. We will compare monthly reminders with reminders implemented every 3 months, and we will compare low intensity, paper-based reminders and high intensity reminders provided by a health care aide peer.

Using interviews, questionnaires, and observations, Sustaining Transfers through Affordable Research Translation (START) will evaluate the processes that inhibit or promote the mobility innovation’s sustainability among health care aides in daily practice. We will examine how the reminders are implemented and perceived by health care aides and licensed practical nurses, as well as how health care aides providing peer reminders are identified, received by their peers, and supported by their supervisors.

**Discussion:**

START will connect up-to-date innovation research with the practice of health care aides providing direct care to a growing population of older Albertans. The project’s reach extends to both supportive living and long-term care settings. Furthermore, START has the potential to introduce and sustain a broad range of innovations in various care areas, such as dementia care, wound care, and pain management – domains where the uptake and sustainability of innovations also encounter significant challenges. By identifying the optimal frequency and intensity of knowledge translation interventions, we hope to enable continuing care organizations to efficiently integrate care innovations into the day-to-day care of residents.

**Trial registration:**

ClinicalTrials.gov, NCT01746459

## Background

Thirty to 40% of patients across all sectors do not receive health care based on current research evidence [1]. This problem is most prominent for those aged 65 years and over, because this is the fastest growing segment of the population among countries in the Organisation for Economic Co-operation and Development (OECD) [2]. It is estimated that only a small proportion of care for older adults is based on research evidence: 29% for urinary incontinence, 35% for cognitive impairment, and 34% for falls and mobility disorders [3]. Leveraging the health benefits of research for older adults is critical when considering their rising number. Closing the gap between research and practice is a pivotal strategy for optimizing health service delivery and health outcomes in the health care system.

Although research is needed to improve the adoption of research innovations, less research has focused on the sustainability of research innovations [4,5]. Sustainability is defined as the extent to which an innovation continues to be used after initial efforts to secure its adoption are complete [6]. Resources invested to introduce a new practice are wasted if adoption of the innovation is transient. The paucity of research in the area of innovation sustainability may be in part due to the tension between sustaining original innovations versus adapting them to local contexts (for example, units with different values, beliefs, and training levels, or exposure to new research programs). Furthermore, the longitudinal data collection required for sustainability research is expensive [5]. To date, our multidisciplinary collaborative research team has studied the effect of the adoption of a research-based mobility innovation on client outcomes through the Canadian Institutes of Health Research (CIHR)-funded demonstration project: the Mobility of Vulnerable Elders (MOVE) study [7]. Now, our team will build upon this study by examining the effect of knowledge translation interventions on the sustainability of health care aide uptake of the MOVE study’s mobility innovation: the sit-to-stand activity. OECD data from 2011 indicates that increasingly populations of older adults are met with high numbers of long-term care staff [8]. Developing inexpensive knowledge translation interventions targeting the health care aides working in supportive living facilities will increase the likelihood that the significant resources invested in promoting the uptake of research will lead to sustained practice change [5] and, ultimately, improved client outcomes.

Knowledge translation interventions are one means to facilitate behavioral change. Several reviews of knowledge translation interventions exist for health care settings [4,9-12]. In the expansive review by Grimshaw *et al*. (2004) of knowledge translation interventions, reminders were the most frequently evaluated, yielding moderate improvements in care and performance [11]. We conducted a secondary analysis of the articles identified in the Grimshaw *et al*. systematic review that focused on reminder systems (n = 42); of these, 26.2% were in acute care settings and 73.8% were in primary care or outpatient settings; none were in continuing care settings. Of these same articles, 21.4% had reminder systems targeting patients, 64.3% targeting physicians, and 14.3% targeting clinicians, including registered nurses, licensed practical nurses, or physician assistants; none of the articles examined reminder systems targeting health care aides. In fact, knowledge translation interventions in continuing care settings are understudied [11,13-16]. A recent scoping review of knowledge translation research in elder care discovered that 3.6% of studies focused on older adults and only 1.8% of these were conducted in continuing care settings [14]. Effective methods to support the sustainability of evidence-based care approaches are especially needed in the continuing care sector where unregulated workers are underappreciated [17] and have limited education yet provide the majority of direct care [18]. Moreover, a systematic review of knowledge translation interventions by Powell *et al*. (2012) concluded that understanding the frequency, intensity, and fidelity of interventions is an important next step in the field of implementation science [19].

Sustaining Transfers through Affordable Research Translation (START) is a randomized controlled trial (RCT); its purpose is to study the effectiveness of reminders to support the sustainability of an affordable mobility innovation by health care aides in supportive living facilities. It will determine which combination of frequency and intensity of reminders most effectively sustains the sit-to-stand activity. Specifically, it will look at peer-based and paper-based reminders, which we will elaborate upon below. This project will help identify how to strike a balance between the desired effects of an innovation and the resources invested to bring them about. START is a collaborative research project that will link interdisciplinary researchers with end-knowledge users involved in policy, advocacy, practice, and education, in striking such a balance. Importantly, this project will bridge the research-to-practice gap by examining the effectiveness and efficiency of reminder interventions to support the sustainability [5] of a research-based mobility innovation (the sit-to-stand activity) [7,20]. A reminder is defined as patient- or encounter-specific information that is provided verbally, on paper, or on a computer screen; such reminders are designed to prompt a health professional to recall information, which would usually be encountered through their general education, in medical records, or through interactions with peers, and subsequently remind them to perform the appropriate care based on up-to-date evidence [21].

START will examine two specific knowledge translation reminder interventions: 1) paper-based reminders; and 2) peer-based reminders. In the absence of literature to guide us about the frequency and intensity of reminders, we decided how to operationalize the low and high levels of frequency and intensity in consultation with collaborative members. For frequency, a monthly modification of the reminders for the high frequency arm aligns with the rhythm of other monthly managerial responsibilities; in contrast for the low frequency reminder, we decided that every 3 months would be infrequent but would align with quarterly managerial responsibilities. For intensity, it was agreed that paper-based reminders are low intensity and commonly used in clinical settings [10]. For the high intensity reminder a socially-based ‘peer reminder’ was favored. Peer reminders have not been reported in the literature; however, we did find one article reporting the use of health care aide champions in a long-term care facility [22] and a protocol trialing a socially-based intervention to move fall prevention evidence into long-term care practices [23]. Media richness theory suggests that the richness of the medium should be selected to fit the nature of the message (in this case a non-routine change in health care aide practice). For example, face-to-face communication with a peer providing reminders (rich medium) offers the possibility of handling multiple information cues, providing rapid feedback, and establishing a personal focus [24]. Compared with a paper-based reminder, which is on the lower end of the media richness hierarchy, the peer reminder might be expected to be more effective in supporting a practice change. Social influence theory emphasizes that behavior is ‘guided… by assumptions, beliefs, and values held by peers and by prevailing practices and social norms that define appropriate behavior’ [25]. Thus, in developing a strategy to promote the uptake of a specific evidence-based practice, the social influence of peers can be leveraged to influence the behavior of health care aides. Our team has experience with paper-based and peer reminders in the MOVE study, but in that study we did not measure health care aide uptake outcomes. (Resident outcomes were measured). In our experience, paper-based reminders were easily introduced, but the peer reminders required more time and effort to implement. Anecdotally, the health care aides providing the reminders appreciated the recognition they received from their managers and experienced satisfaction in their roles. To summarize, the level of reminders will vary in frequency (monthly versus every 3 months) and intensity (paper reminders versus paper reminders plus peer reminders).

We will also study facility context [26,27] and processes that influence the effectiveness of reminders to support the ongoing uptake of the activity by health care aides in 24 supportive living facilities.

### Theoretical/conceptual framework

**Figure 1:**
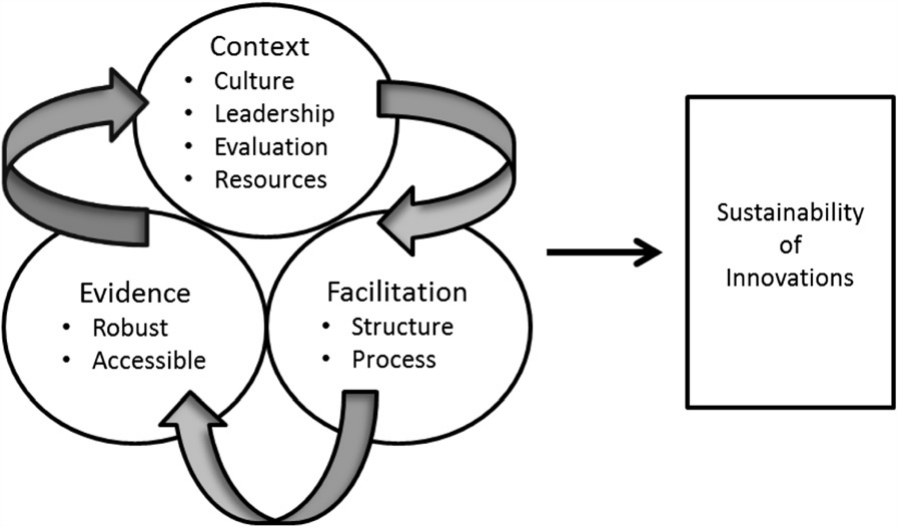
**Conceptual framework for START project.** Adapted from the PARIHS framework [28]. PARIHS, Promoting Action on Research Implementation in Health Services; START, Sustaining Transfers through Affordable Research Translation.

This research is guided by the Promoting Action on Research Implementation in Health Services (PARIHS) conceptual framework [28], which posits that successful implementation of evidence into practice involves connections between facilitation [29], evidence [30], and context [31] (Figure 1).

If these three domains are ‘strong’, then there is an increased likelihood that evidence will be adopted into practice. The PARIHS framework accounts for the complex and multilevel nature of initial adoption of innovations in long-term care settings [32]. Key concepts in the PARIHS framework are similar to concepts identified as important for the sustainability of innovations in two systematic reviews [5,9]. We have adapted the PARIHS framework to include the sustainability of innovations. Facilitation involves structures and processes that enable individuals, teams, and organizations to change [29]. The facilitation processes in the current study pertain to the use of reminders as a type of knowledge translation intervention. Evidence is defined as knowledge derived from a variety of sources that has been tested and deemed credible [30]. The evidence to be integrated into practice for the START project is the sit-to-stand activity that has been studied with older adults in both laboratory and clinical settings. Context is defined as the environment in which people receive healthcare services, and in which the proposed change is to be implemented [31]. START will examine factors that the PARIHS framework identifies as foundational to a facility’s context (the prevailing culture, leadership roles, and how evaluation is conducted) in supportive living facilities in a western Canadian city. We will describe how the key concepts of the PARIHS framework relate to START in the following paragraphs.

#### Facilitation

Given the novel nature of the high intensity intervention (peer reminders), we will use educators to conduct focus groups with health care aides before starting the clinical trial to further develop this knowledge translation intervention. These focus groups will address how to best tailor the peer reminders for the participating facilities. We will ask health care aides to consider the proposed reminder, and ask participants to brainstorm possible ways to operationalize the reminder system. During the focus groups we will ask health care aides to evaluate and discuss the merits of their proposed ideas, as well as ideas suggested by the lead educator. Possible examples of the peer reminder may include: 1) discussing case studies; 2) discussing documentation; 3) discussing sticker reminders; 4) describing ‘good news stories’; and 5) peers celebrating other peers.

The success of the health care aide peer reminder role hinges on achieving the right fit between the role and the health care aide recruited to the role. Experienced health care aides who have established working relationships with their peers and health team members will contribute to the credibility of the peer reminder role. Health care aides are more likely to feel comfortable in the peer reminder role if they demonstrate the ability to: influence others; attract respect from peers, residents, and professional staff; exhibit effective communication skills; show enthusiasm for new practices; and demonstrate a passion for their role as a caregiver. It is likely that the peer reminder health care aides will have experience championing other new practices in their facilities, such that their mentorship skills may be more developed. Ideally the peer reminder health care aides will work full-time and on both day and evening shifts.

It is important to note that paper-based reminders are common; the studies in the Grimshaw *et al*. review largely used paper-based reminders in primary care settings [11]. Reminders with a paper-based component were also most frequent in a review exclusively examining physician reminders [10]. Paper-based reminders worked well when few care measures were involved and when the reminders were integrated into the clinical workflow. However, peer-based reminders are a novel intervention and, to the best of our knowledge, no study has attempted to use this intervention to support the sustainability of an innovation. Thus, the focus groups will help guide, formulate, and thus facilitate an appropriate, feasible peer-based reminder for health care aides working in continuing care facilities.

#### Evidence

We decided to study the sustainability of the sit-to-stand activity because it possesses most of the attributes of an innovation that are thought to increase the likelihood of successful adoption [33], and research evidence supports the sit-to-stand activity’s effectiveness in maintaining mobility. Rogers’ attributes of a successfully adopted innovation include: 1) relative advantage (requires minimal training of health care aides or clients); 2) compatibility (builds upon existing routines); 3) low complexity (low cost innovation conducted by regular staff; does not involve an important increase in the time required to care for clients); 4) trialability (easily tried and adapted to individual clients); and 5) observability (outcome can be visible) [6]. Process data from the MOVE study suggest that health care aides can integrate the sit-to-stand activity into their care routines (for example, during dressing and toileting) [7]. Preliminary outcome data from 70 MOVE study participants found that older adults who engaged in the sit-to-stand activity for 3 months were 1.35 times more likely to improve in their ability to transfer than participants not doing the activity. Our evidence extends that of others suggesting that performance of the sit-to-stand activity can delay the well-known trajectory of functional decline in continuing care residents [34-37]. However, unless the activity is consistently performed, it cannot delay this decline.

#### Context

The context in which an innovation is deployed is as important a determinant of the adoption and sustainability of the innovation as the innovation itself [5,9]. Although previous research has conceptualized innovation adoption as a discrete decision and focused on an outcome of interest, systematic reviews on organizational context emphasize its importance in understanding how and why innovations are adopted and assimilated into clinical practice [9]. The organizational context of the START study is the emerging sector of supportive living facilities in Alberta, which has expanded significantly since the introduction of Alberta’s Continuing Care Strategy [38]. These facilities generally employ 24-hour on-site health care aides and a licensed practical nurse. Usually a registered nurse is available on-call 24 hours a day. In addition, facility-based case managers develop care plans and monitor the care provided. One of the few studies conducted in this sector was a 1-year cohort study that identified the health and social needs of clients, the mix of services provided, and health outcomes [39]. Of the 1,089 supportive living participants in the study, the majority were independent in walking (59%) and transferring (76%), while only 42% were independent in activities of daily living [40]. A third of the study participants either died or moved to residential long-term care facilities during the year of follow-up, pointing to the risk of rapid decline in mobility and activities of daily living in the supportive living population.

Prior to randomization we will assess the equivalence of the participating supportive living facilities by measuring organizational context using the Alberta Context Tool (ACT) [26]. This instrument includes modifiable dimensions of organizational context that could influence the use of new knowledge including: culture, leadership, evaluation, social capital, informal interactions, formal interactions, structural and electronic resources, and organizational slack (three sub-concepts: time, space, and human resources).

To study the effectiveness of reminders to support the sustainability of an affordable mobility innovation by health care aides in supportive living facilities, we will address the following research questions.

### Research questions

1. Do more frequent reminders (every month) improve the sustainability of the sit-to-stand activity by health care aides in supportive living facilities compared with less frequent reminders (every 3 months)?

2. Do high intensity reminders (paper-based reminders plus peer reminders) improve the sustainability of the sit-to-stand activity by health care aides in supportive living facilities compared with low intensity reminders (paper-based reminders only)?

3. Do more frequent reminders plus high intensity reminders synergistically improve the sustainability of the sit-to-stand activity by health care aides in supportive living facilities?

4. What are the processes associated with the ongoing uptake of the sit-to-stand activity over a year of follow-up?

### Hypotheses for research questions 1 to 3

1. Arm 1 with low intensity and low frequency reminders will have poor uptake of the activity.

2. Arm 2 with low intensity and high frequency reminders will have moderate uptake of the activity.

3. Arm 3 with high intensity and low frequency reminders will have moderate uptake of the activity.

4. Arm 4 with high intensity and high frequency reminders will have excellent uptake of the activity.

## Methods/Design

START will conduct a phase III, multicentre, cluster RCT [41,42] using a stratified 2 × 2 additive factorial design in 24 supportive living facilities within the health zone of Edmonton, AB, Canada (research questions 1 to 3), and will include an embedded process evaluation [43,44] (research question 4). Through this design, we will determine which combination of frequency and intensity of reminders is required to efficiently and effectively sustain the sit-to-stand activity. The four treatment arms that combine frequency and intensity of reminders are: 1) low intensity and low frequency; 2) low intensity and high frequency; 3) high intensity and low frequency; and 4) high intensity and high frequency.

The advantage of using a factorial design over a parallel-group design is that the features of two knowledge translation interventions (that is, frequency and intensity) can be examined simultaneously in the same group of participants (thus reducing the sample size by one-half). It also allows for an assessment of a potential synergistic effect between the two intervention features. Cluster randomization is being used because the interventions are administered at the facility level, and it is impossible to randomize individual health care aides (or their respective units) without contaminating the intervention arms with cross-talk among aides.

### Project plan

**Figure 2:**
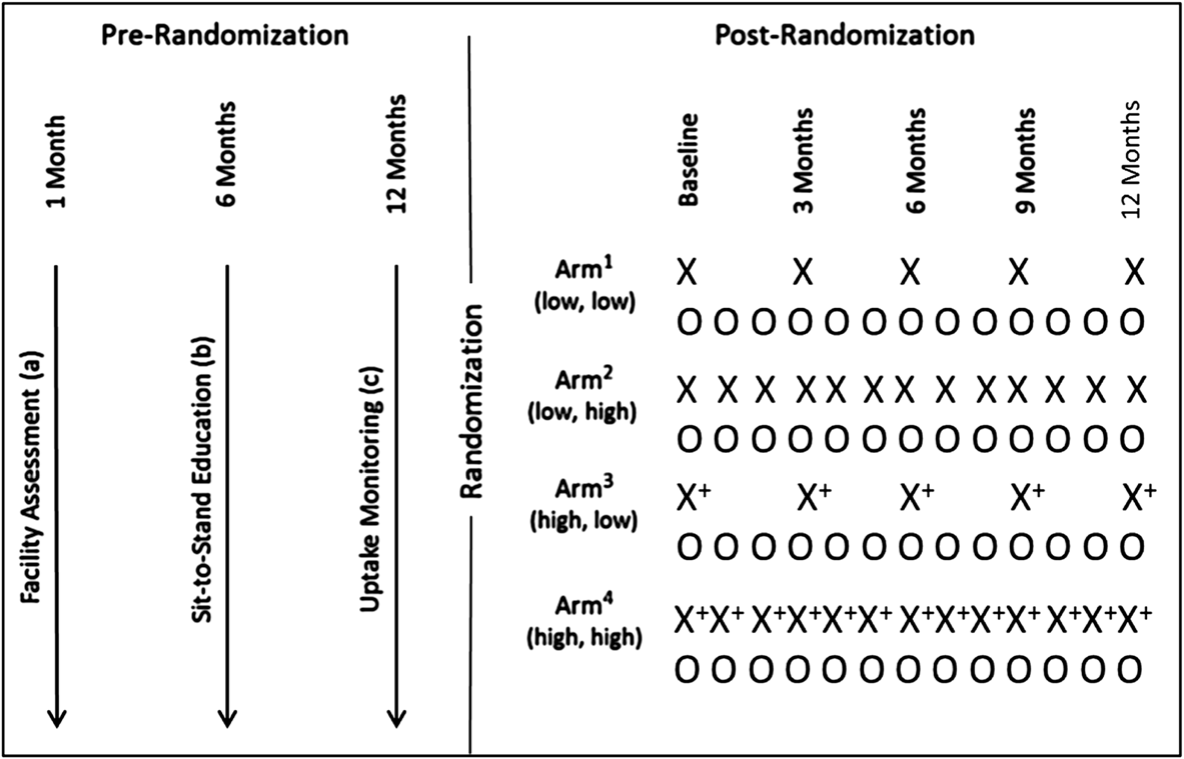
**Randomized controlled trial (RCT) in a factorial design.** O, measurement of uptake; X, paper-based reminders (low intensity); X^+^, paper-based reminders plus peer reminder (high intensity).

The project plan includes details of the pre-randomization, randomization, and post-randomization phases of the trial, the measures used, and the process evaluation (Figure 2).

### Pre-randomization

The purpose of the pre-randomization period is to prepare for randomization by: 1) facility assessments to identify three ‘equivalent’ groups of facilities to be randomized across the arms; 2) sit-to-stand education sessions to establish initial adoption of the mobility innovation; and 3) uptake monitoring to ensure the primary outcome measure, uptake of the innovation, is operational in each facility.

#### Facility assessment

Eligible facilities will have a minimum of 30 designated supportive living beds in the Edmonton zone. In the first 6 months, we will conduct a facility assessment with 24 potential facilities using the ACT [45] to compare organizational context, and the work and well-being survey [46,47] to compare health care aide work engagement. Data from this multilevel assessment will be used to stratify the facilities into three ‘equivalent’ groups for randomization.

#### Education sessions

Concurrent with client recruitment, the study educator (in collaboration with the facility-based educator) will use 20-minute education sessions to train health care aides working day and evening shifts to complete the sit-to-stand activity. They will deliver these sessions a minimum of four times on participating units. The standardized education protocol (Additional file 1) was developed for this purpose by our education partner and evaluated by the research team. In brief, these interactive education sessions: 1) describe the benefits of the sit-to-stand activity for both clients and health care aides; 2) demonstrate the activity; 3) explain how the paper-based reminders will identify the clients participating in the activity; and 4) review documentation of client participation with the sit-to-stand activity using flowsheets. Health care aides will be taught (and expected by their employer) to prompt participating clients to slowly and repeatedly stand up and sit down on four occasions: twice on each day and evening shift. The number of repetitions on each occasion will vary with clients’ ability and fatigue. Health care aides will integrate the sit-to-stand activity into usual care routines (for example, while dressing or toileting) on two occasions during their shift.

#### Uptake monitoring

On the first day of the month following the completion of the education sessions, the manager or nurse in charge will announce the beginning of the sit-to-stand activity during report for the morning and evening shift changes. A list of participants posted at the shift change location will inform the health care aides about who is participating in the sit-to-stand activity. Aides will carry out the activity with participants in their care assignment, and record the number of sit-to-stands completed during each occasion on the flowsheet. Flowsheets for each participant will be collected at the end of every month. To optimize the fidelity of the innovation [42] leading to the secondary outcome measure (client mobility), we will monitor uptake and enhance the reliability of the health care aides’ flowsheet documentation for 2 months prior to randomization. First, the study educator will conduct two informal walkabouts with staff at each facility to discuss the activity and clarify any misconceptions. Second, on 3 days within the first week of each of the 2 months, a research assistant will review the documentation flowsheets by correcting and noting proper documentation style directly on the flowsheets. Third, a research assistant will conduct two documentation flowsheet information sessions at each facility, providing sample flowsheets and discussing with health care aides the correct documentation procedure.

### Randomization and client recruitment

A stratified blocked randomization procedure will assign facilities to intervention arms. Each of the three strata consisting of eight ‘equivalent’ supportive living facilities will be randomized separately to four intervention arms by first constructing two blocks of four facilities within each stratum. Using computer-generated random numbers, facilities within each block will be randomized to one of the four intervention arms. Block randomization ensures that an equal number of ‘equivalent’ facilities are assigned to each of the four arms. Research assistants will recruit clients to participate throughout the study with replacement when a client moves or dies. Clients will be eligible to participate if they can transfer independently or with the assistance of one person.

#### Blinding

Team members will be blinded to the four intervention arms with the exception of the research manager and the staff assigned to implement the reminder interventions. Research assistants implementing the reminders will be instructed to avoid discussing their work with the research team.

### Post-randomization

Immediately following randomization, a simple set of paper-based reminders will be introduced to all sites to heighten health care aides’ awareness of the sit-to-stand activity. The low intensity paper-based reminders that were tested in the MOVE study and are commonly used in clinical settings to introduce practice change, include: 1) affixing stickers to clients’ bedroom doors, beside their beds, or in their bathrooms (Additional file 2); 2) posting signs in prominent locations (Additional file 3); and 3) placing colored flags on the documentation flowsheets. Every month for the high frequency sites, and once every 3 months for the low frequency sites, the color or shape of the paper-based reminders will be modified. For the high intensity reminders, health care aides will be identified to offer peer reminders based on their demonstrated informal leadership behavior during the education sessions and in consultation with their managers. These health care aides will provide formal and informal peer reminders about the sit-to-stand activity; the formal reminders will take place either monthly or every 3 months during change of shift meetings, while the informal reminders will be provided as opportunities arise during the work day. Every month for the high frequency sites and every 3 months for the low frequency sites, the study educator will coach the health care aides providing peer reminders. To optimize the fidelity of the reminder interventions [42] leading to the primary outcome measure (health care aide uptake), a committee will be established to monitor the fidelity of the reminder interventions and recommend strategies to mitigate any identified problems.

### Measures (research questions 1 to 3)

#### Primary outcome measure

As the purpose of this study is to examine the effectiveness of reminders to support the sustainability of a mobility innovation by health care aides, the primary outcome is health care aide uptake as operationalized by the number of completed sit-to-stand occasions. In the absence of a previously developed uptake measure [48], we have validated a documentation flowsheet (Additional file 4). Health care aides will record on this flowsheet the number of sit-to-stands that the client completes on each of two occasions on the day shift and on the evening shift (that is, four occasions per day). Research staff will score the flowsheet for each occasion with ‘1’ denoting a completed occasion of sit-to-stand activity and ‘0’ denoting that the sit-to-stand activity was not completed.

The reliability and validity of these documentation flowsheets were assessed using two methods. First, 31 health care aides viewed two videotaped vignettes depicting the sit-to-stand activity, and recorded on flowsheets the number of sit-to-stands the clients performed. The exact agreement between observed and recorded number of sit-to-stands was 90.3% for the first vignette and 80.6% for the second vignette. Second, we used logistic regression to examine the relationship between the change in 26 clients’ mobility and the number of occasions that the sit-to-stand activity was completed (odds ratio = 1.07; *P* = 0.023) to assess concurrent validity. Clients that completed the sit-to-stand activity more often, as recorded on flowsheets, were more likely to either maintain or improve their ability to stand up from a chair when compared with clients that completed the activity less often.

In addition to this completed validation work, we have piloted two other validation approaches. Preliminary results from observing five clients show 87.5% exact agreement between the number of observed and recorded sit-to-stands. We also used the activPAL3 activity monitoring device (Pal Technologies, Glasgow, UK), which provides minute-by-minute information on the client’s activity. ActivPAL counts of standing and sitting repetitions in stroke patients has good exact agreement with counts by direct observation, an indication of concurrent validity [34,49]. Preliminary results using the activPAL3, to compare activity output of five clients with flowsheet records for 3 days, show 84.6% exact agreement. There was no evidence of skin irritation, and clients forgot they were wearing the device.

START will continue to assess the reliability of the flowsheets across all four treatment arms. We will compare direct observations of 28 clients (seven in each of the four treatment arms) completing the sit-to-stand activity with the number of sit-to-stands recorded by health care aides on flowsheets. Similarly we will compare the activPAL3 output of 28 clients with their flowsheet recordings. A sample size of 27 subjects yields 80% power to demonstrate excellent agreement (correlation = 0.85), assuming 0.95 for the true correlation.

#### Sample size calculation for primary outcome

**Figure 3:**
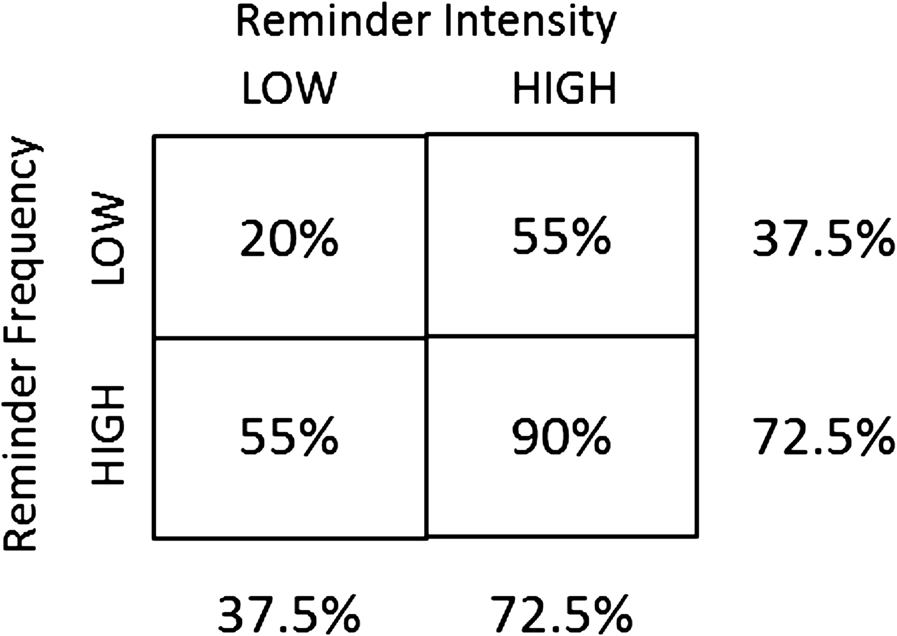
Sample size assumptions.

The required sample size for the trial is 24 facilities, each with an average of 15 health care aides assigned to two clients. Each aide is expected to have 1,440 (2 × 2 × 30 × 12) possible uptake occasions with each client, for an expected total of 43,200 (15 × 2 × 1,440) aide-occasions per facility over the trial’s duration. The sample size calculation, based upon previous results from four facilities consisting of 44 aides [7,50], yielded an estimated non-interventional uptake rate of 22.6% with a coefficient of variation (CV) of 0.54 among facilities. The CV plays the role of the between-facility clustering parameter in the sample size formula for rates [51]. For the purpose of sample size calculations, we assume the low-low arm to have an uptake rate of 20%, the single-high arms to have an uptake rate of 55%, and the double-high arm to have an uptake rate of 90%. The additive trial design has 80% power at a 5% two-sided level of significance to detect a 93.3% relative increase in the marginal rates of uptake (72.5% versus 37.5%) between high and low (Figure 3).

#### Secondary outcome measure

We will also measure the sustainability of client mobility across the four intervention arms. At the end of a year of data collection, a sample of clients will be assessed using the 30-second sit-to-stand test. The sit-to-stand action is a functional activity that has been incorporated into a number of mobility measures including the time for five sit-to-stands and the number of sit-to-stands completed in 30 seconds (the 30-second sit-to-stand test). We will measure the 30-second sit-to-stand test because in our population clients may be unable to complete more than two or three sit-to-stands [37,52-54]. Using a stopwatch and a standard armchair, we will instruct client participants to stand up and sit down as many times as possible until they are asked to stop after 30 seconds. In community-dwelling older adults, there is evidence for test-retest reliability, criterion validity (chair stand performance compared with lower body strength), and discriminant validity (performance of different age and physical activity groups) with this 30-second sit-to-stand test [52].

#### Sample size calculation for secondary outcome

We expect 65% of the original 720 clients to survive, yielding 468 clients. A proposed sample size of 200 clients (50 per arm) will have 80% power at a 5% two-sided level of significance to detect an absolute mean difference of one sit-to-stand (5.5 versus 4.5) between high and low treatment arms. The calculation, based upon results from six facilities of 75 clients from the MOVE study, yielded an estimated non-interventional mean number of 4.3 (SD = 2.5) sit-to-stands with an intraclass correlation coefficient (ICC) of −0.05 within each facility. For the purpose of sample size calculation, we assume at 1-year follow-up, the low-low arm will have a mean number of sit-to-stands of four on the 30-second sit-to-stand test, the low-high and the high-low arms will have a mean number of five, and the high-high arm will have a mean number of six.

#### Pre-randomization facility assessment measures

Characteristics of supportive living facilities, health care aides, and clients will be used to describe the sample, identify ‘equivalent’ facility groups for block randomization, and add to the process evaluation.

Facility/unit characteristics include facility and unit size (bed number), facility ownership model (for-profit or not-for-profit), client-to-staff ratios, rehabilitation service availability, and organizational context (ACT) [26]. It is a reliable and valid measure of organizational context when completed by individual care providers in pediatric units [45,55] or nursing homes [56,57]. A total of 15 health care aides will complete the ACT in each participating facility. Eligible health care aides must work a minimum of six shifts per month and have worked at least 3 months in the facility.

Health care aide demographic characteristics (age, sex, education, first language, duration of employment) will be assessed, and the 9-item Utrecht Work Engagement Scale (UWES) (Additional file 5) [46,47] will be completed when the ACT is administered. The UWES assesses the work engagement dimensions of vigor, dedication, and absorption.

### Trial data management and analysis (research questions 1 to 3)

Data will be entered by a contracted agency into a statistical database (SPSS version 19; IBM, Armonk, NY, USA) programmed to minimize data entry errors. All of the data will be double entered to assess the accuracy of data entry. Research participants will be assigned unique identifiers and all personal identification will be removed before data cleaning and analysis. A graduate student will be hired to clean the data using standard data management techniques. All analyses will follow the intention-to-treat principle, namely that all participants will be included regardless of deviation from protocol. SAS 9.2 (SAS Institute, Cary, NC, USA) software will be used to perform the aide-level analyses. For the primary outcome measure, the total number of completed sit-to-stand occasions out of the total number of possible (completed and not completed aide-occasions) will be calculated. As the design is a stratified additive 2 × 2 factorial, generalized estimation equations (GEE) with a log link, Poisson distribution, an exchangeable working correlation structure, and no interaction term will be used to simultaneously test the significance of the high versus low intensity and frequency using Wald tests (PROC GENMOD). The model will include ‘equivalent’ strata as a covariate and log total aide-occasions as the offset. Robust variance estimators will be employed to adjust for the effect of clustering. Although the size of the trial is not statistically powered to detect a synergistic effect between intensity and frequency, an interaction term will be incorporated into the model to test for this effect. In the event that imbalance on baseline facility and health care aide characteristics among groups occurs, GEE naturally facilitates additional analyses to examine, and control for, their influence. Rate ratios will be used in quantifying the effectiveness of the high versus low intensity and frequency reminders. For the secondary outcome measure, a mixed effects linear regression model (PROC MIXED) will be used, with facility considered as a random effect to account for the clustering, no interaction term, and ‘equivalent’ strata and baseline sit-to-stand number as covariates. An interaction term will be incorporated into the model to test for a synergistic effect between intensity and frequency, as well as baseline facility and health care aide characteristics if unbalanced. Least squares means will be used to quantify the effectiveness of the interventions. Based on the Medical Research Council (MRC) framework for developing and evaluating complex interventions, we are moving from the pilot stage to the evaluation stage of knowledge translation interventions [43].

### Process evaluation (research question 4)

The goal of the process evaluation is to understand how facility processes and reminders affect the sustainability of health care aide uptake of the sit-to-stand activity. This evaluation will enable an understanding of: unexpected outcomes, fidelity of the interventions (including local adaptations of the interventions) [44], ‘active ingredients’ of the four intervention arms [58], subgroup variation, and the influence of contextual factors on outcomes [43,44,59]. We will examine how the reminders are implemented and perceived by participants, as well as how health care aides providing peer reminders are identified, received by their peers, and supported by their supervisors. Key components of this evaluation involve a careful analysis of potential barriers and facilitators that inhibit or promote practice change, the need to refine the reminders, and the value of extending their use to other supportive living facilities. Process evaluations are indicated in multisite trials where the ‘same’ intervention may be implemented and received differently across sites [59].

To augment the standardized measures from the facility assessments, we will collect further process data using observations, questionnaires, and interviews. For observations, anytime that research staff enter a study facility, they will be alert to observe responses of facility staff or clients to the reminder interventions. Upon exiting the facilities, they will immediately record fieldnotes [60]. Organizational processes will be especially evident when collaborating with facilities to recruit clients to the study and collect monthly flowsheets. Educators’ fieldnotes will be useful for understanding processes of and responses to coaching the health care aides that provide reminders. For the questionnaire, we will survey a licensed practical nurse and a manager from each facility using a questionnaire to elicit perceptions of the reminders (see Additional file 6). For interviews with health care aides, to understand health care aides’ views of the reminders, we will use interviews rather than written questionnaires, as many health care aides speak English as a second language (see Additional file 7). We will interview approximately six health care aides from purposively sampled facilities until we achieve saturation [61]. Four facilities (two positive and two negative extreme cases; one from each arm) will be sampled based on facility assessments. For interviews with peer reminders, to understand the peer reminder experience, we will interview approximately six health care aides providing peer reminders from each high intensity reminder arm until saturation (see Additional file 8).

Text from fieldnotes, questionnaires, and interviews will be imported into ATLAS.ti (ATLAS.ti Scientific Software Development, Berlin, Germany), and checked for accuracy. The data will be coded, classified, and analyzed thematically based on interpretive description principles to generate a description that informs understanding [62]. This analysis, along with the facility assessments, will allow us to describe, compare, and generate hypotheses about the processes associated with the sustainability of the sit-to-stand activity across the four intervention arms [59].

### Expected outcomes

Recognizing the need to balance the effectiveness of the innovation’s sustainability with efficient resource allocation, this study will identify the frequency and intensity of reminders required to sustain the behavior of health care aides to complete the sit-to-stand activity. At the end of the study we will know: 1) if less frequent reminders are as effective as more frequent reminders in sustaining the health care aides’ behavior; 2) if the peer reminders added to the paper-based reminders are more effective than paper-based reminders alone in sustaining the health care aides’ behavior; 3) if more frequent paper-based reminders plus the peer reminders synergistically improve the sustainability of the health care aide’s behavior; and 4) how facility processes and the reminders are associated with the sustainability of health care aides completing the sit-to-stand activity.

### Ethical considerations

We received ethical approval for the study from the Health Research Ethics Board at the University of Alberta, Edmonton, AB, Canada.

#### Client consent

Research assistants will obtain written informed consent to obtain clients’ health records and baseline mobility directly from clients that have the capacity to consent to research. They will obtain consent from authorized representatives for clients lacking capacity to consent. The geriatrician co-leader will train research assistants to assess capacity to consent to research. Assent of clients will be assessed by their willingness to have their baseline mobility measured by a research assistant.

#### Health care aide consent

Research assistants will obtain written informed consent from health care aides before interviews or questionnaires.

#### Facility consent

We will seek a letter of support from each facility that includes a statement of acceptance of the study’s organizational impact and agreement for the sit-to-stand activity to be an organizational expectation.

## Discussion

Through the participation of the collaborative members and partners, the START project will connect innovative implementation research with continuing care practice, health care aide education, provincial advocacy, and health policy. Identifying effective and efficient reminders to maintain evidence-based innovations will increase the likelihood that resources invested to introduce innovations are sustained in practice. This, in turn, can lead to improved health outcomes for this growing population of vulnerable older adults. The collaborative members of the team are well-positioned to inform and assist with translating the study findings, and we expect to be able to spread these tested reminders to other supportive living facilities. Furthermore, the project’s reach may extend to a broad range of care domains, such as pain, falls, end-of-life, and dementia – domains where the sustainability of innovations can also encounter significant challenges. We expect the results of this cluster RCT to contribute to sustainable innovations in the continuing care sector and, in particular, to the sustained use of an affordable mobility innovation in supportive living settings.

## Trial status

We have only begun to recruit facilities (2 of 24 facilities) and health care aides (35 of 360 health care aides). We have not begun to recruit any older adults to the study. We expect to recruit 720 residents from the facilities.

## Abbreviations

ACT: Alberta context tool; CIHR: Canadian Institutes of Health Research; CV: Coefficient of variation; GEE: Generalized estimation equation; ICC: Intraclass correlation coefficient; MOVE: Mobility of vulnerable elders; MRC: Medical Research Council; OECD: Organisation for Economic Co-operation and Development; PARIHS: Promoting Action on Research Implementation in Health Services; RCT: Randomized controlled trial; START: Sustaining Transfers through Affordable Research Translation; UWES: Utrecht work engagement scale.

## Competing interests

The authors declare that they have no competing interests.

## Authors’ contributions

SES conceived of the study, led in the development of the protocol, and prepared the initial draft of the manuscript. CAE, CAJ, AW, and ME participated in the development of the study design and revisions of the protocol. All authors read and approved the final manuscript.

## Authors’ information

SES is Assistant Professor, Faculty of Nursing, University of Alberta. SES holds a New Investigator Award in Chronic Disease from the Canadian Nurses Foundation and the Canadian Institutes for Health Research. CAE is Professor, Faculty of Nursing, University of Alberta. CAE holds a Canadian Institutes for Health Research, Canada Research Chair in Knowledge Translation. CAJ is Associate Professor, Faculty of Rehabilitation Medicine, Department of Physical Therapy, University of Alberta. CAJ holds a Canadian Institutes for Health Research, New Investigator Award and an Alberta Heritage Foundation for Medical Research, Population Health Investigator Award. AW is Professor of Healthy Ageing, Faculty of Medicine and Dentistry, University of Alberta. AW is the Director of the Division of Geriatric Medicine. ME is Associate Professor, Department of Public Health and Community Medicine, Tufts University School of Medicine.

## Supplementary Material

Additional file 1 Sit-to-stand activity lesson plan excerpt. Click here for file

Additional file 2 Sit-to-stand paper-based reminder examples. Click here for file

Additional file 3 Example reminder system poster. Click here for file

Additional file 4 Sit-to-stand documentation flowsheet. Click here for file

Additional file 5 Work and well-being survey. Click here for file

Additional file 6 Questionnaire for licensed practical nurse and facility leader. Click here for file

Additional file 7 Interview guide for health care aides. Click here for file

Additional file 8 Interview guide for peer reminder. Click here for file
