# Supplementary material for: Sustaining Transfers through Affordable Research Translation (START): study protocol to assess knowledge translation interventions in continuing care settings
Source: Trials. 2013 Oct 26;14:355. doi: 10.1186/1745-6215-14-355 (PMC4231466; doi:10.1186/1745-6215-14-355)
Supplement: Additional file 1 — Sit-to-stand activity lesson plan excerpt. [file 1745-6215-14-355-S1.doc]

Additional file 1

Sit-to-Stand Activity Lesson Plan Excerpt

**Objectives of Sit-to-Stand Education Session (20 Minutes):**

1. Introduce sit-to-stand activity within Healthcare Aide scope of practice;
2. Review safe transfer and body mechanics techniques;
3. Solve potential challenges using various case scenarios
4. Describe related documentation;
5. Describe follow-up communication strategies (monthly knowledge translation interventions)

**Definition:** The sit-to-stand activity is repeating the action of slowly standing up and slowly sitting down.

**Purpose of Sit-to-Stand Activity**: To maintain the ability of clients to transfer independently or with just one-person assist.

**Benefits for Clients:** Maintains clients function for Activities of Daily Living. Client can transfer independently (or with just one-person assist).

**Benefits for Healthcare Aides:** Transfers are safer for staff (less back injuries, for example); uses less resources (don’t require mechanical hoists, only need one Healthcare Aide and not two per transfer.)

**Policy:** To maintain the ability of clients to transfer, all clients who are able to transfer independently or with a one person assist will be encouraged and assisted by Healthcare Aides to practice the sit-to-stand activity twice on the day shift and twice on the evening shift. Every day and evening shift Healthcare Aides will record the number of times clients are able to stand during each occasion that the sit-to-stand activity was performed.

**Procedure:**

1. The client is seated. If a wheelchair is used then the wheelchair is locked and placed with the back against a wall.
2. Following the Facility Transfer Policy, ask the client to slowly stand up and sit down as many times as s/he can.
3. Do this activity two times during the day shift and two times during the evening shift.
4. The activity is completed at a time when clients would already be transferring: for example when they are dressing, toileting or standing up from the dining room table.
5. If the client’s health condition has changed then Healthcare Aides will consult with the nurse in charge about whether or not to do the sit-to-stand activity.

**Documentation:**

- During the day and evening shifts Healthcare Aides will record on a flowsheet the number of sit-to-stands that were completed for each of the two times it was completed.
- If the client did not complete the sit-to-stand activity then a 0 will be entered in the appropriate space for that shift on the flowsheet.
