# Supplementary figures and images for: Sustaining Transfers through Affordable Research Translation (START): study protocol to assess knowledge translation interventions in continuing care settings
Source: Trials. 2013 Oct 26;14:355. doi: 10.1186/1745-6215-14-355 (PMC4231466; doi:10.1186/1745-6215-14-355)

## Slide 1
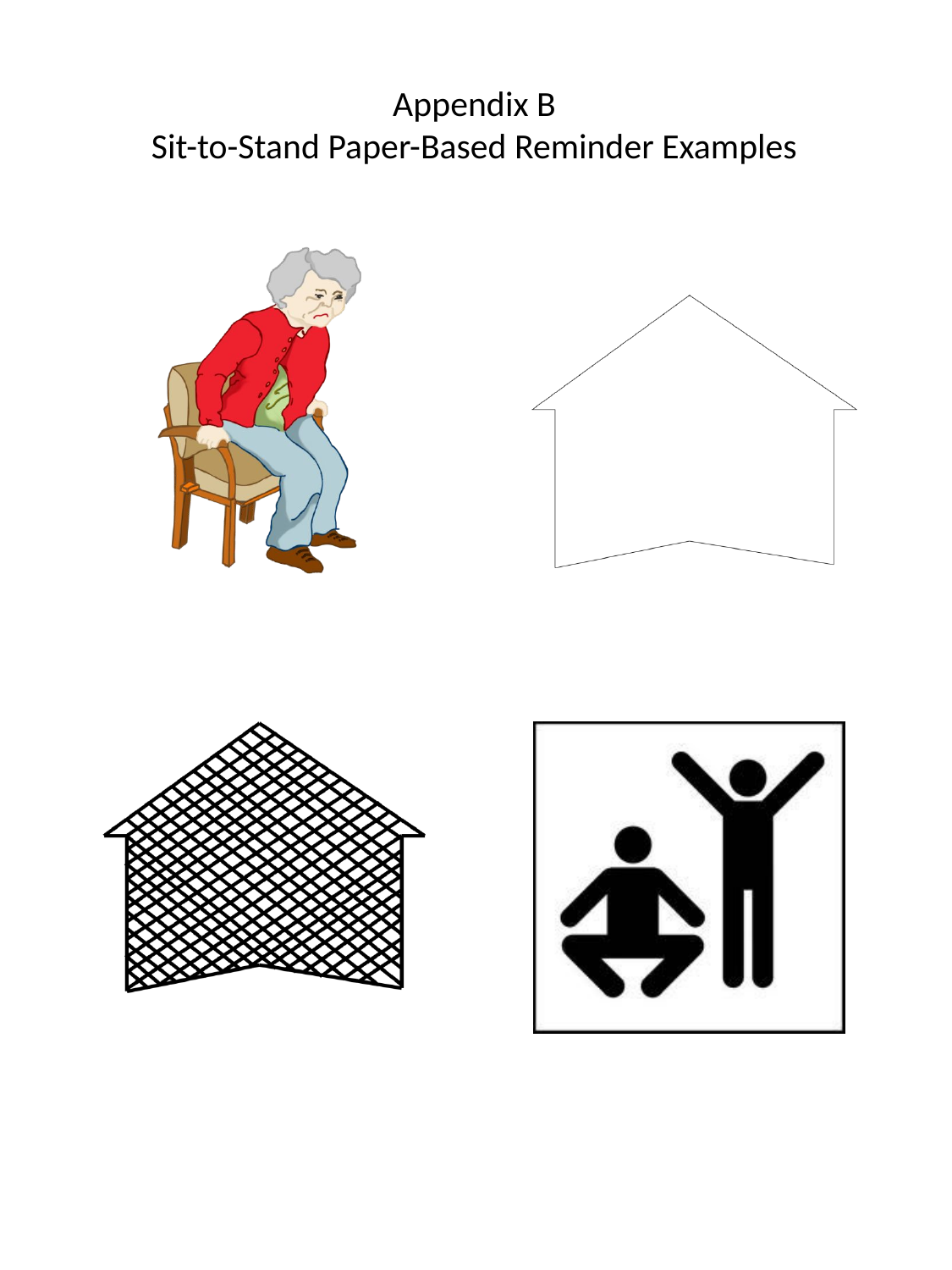

Appendix B
Sit-to-Stand Paper-Based Reminder Examples

Supplement: Additional file 2 — Sit-to-stand paper-based reminder examples. [file 1745-6215-14-355-S2.pptx]
