# Supplementary material for: Sustaining Transfers through Affordable Research Translation (START): study protocol to assess knowledge translation interventions in continuing care settings
Source: Trials. 2013 Oct 26;14:355. doi: 10.1186/1745-6215-14-355 (PMC4231466; doi:10.1186/1745-6215-14-355)
Supplement: Additional file 4 — Sit-to-stand documentation flowsheet. [file 1745-6215-14-355-S4.doc]

Additional file 4

Client Label

Facility Label

Sit-to-Stand Documentation Flowsheet

Please do this activity two times on your shift and write the number of repetitions in the box below.

Please ask the resident to repeat standing up and sitting down.

Please do this exercise twice on your shift.

Please write the number of times the resident stands up and sits down in the box.

When the resident is well, they can do: **5** repetitions + maybe more

Month:

| Dates | 1 | 2 | 3 | 4 | 5 | 6 | 7 | 8 | 9 | 10 | 11 | 12 | 13 | 14 | 15 | 16 | 17 | 18 | 19 | 20 | 21 | 22 | 23 | 24 | 25 | 26 | 27 | 28 | 29 | 30 | 31 |
| --- | --- | --- | --- | --- | --- | --- | --- | --- | --- | --- | --- | --- | --- | --- | --- | --- | --- | --- | --- | --- | --- | --- | --- | --- | --- | --- | --- | --- | --- | --- | --- |
| Day Time 1    Day Time 2  Evening Time 1 | 4  3 | 5  3 | 2  5 | 1  5 | 0  0 | 0  2 | 5  2 | 8  5 | 9  6 | 9  6 | 5  6 |  |  |  |  |  |  |  |  |  |  |  |  |  |  |  |  |  |  |  |  |
| Evening Time 2 | 4  6 | 5  6 | 5  6 | 6  6 | 6  5 | 5  5 | 5  5 | 5  7 | 4  5 | 0  3 | 4  5 |  |  |  |  |  |  |  |  |  |  |  |  |  |  |  |  |  |  |  |  |

Initials

| Initials | 1 | 2 | 3 | 4 | 5 | 6 | 7 | 8 | 9 | 10 | 11 | 12 | 13 | 14 | 15 | 16 | 17 | 18 | 19 | 20 | 21 | 22 | 23 | 24 | 25 | 26 | 27 | 28 | 29 | 30 | 31 |
| --- | --- | --- | --- | --- | --- | --- | --- | --- | --- | --- | --- | --- | --- | --- | --- | --- | --- | --- | --- | --- | --- | --- | --- | --- | --- | --- | --- | --- | --- | --- | --- |
| Day | CI | CI | CI | SS | SS | CI | CI | CI | CI | SS | CI |  |  |  |  |  |  |  |  |  |  |  |  |  |  |  |  |  |  |  |  |
| Evening | AJ | AJ | JL | JL | AJ | AJ | AJ | JL | JL | JL | AJ |  |  |  |  |  |  |  |  |  |  |  |  |  |  |  |  |  |  |  |  |

Comments: ____________________________________________________________
