# Supplementary material for: Sustaining Transfers through Affordable Research Translation (START): study protocol to assess knowledge translation interventions in continuing care settings
Source: Trials. 2013 Oct 26;14:355. doi: 10.1186/1745-6215-14-355 (PMC4231466; doi:10.1186/1745-6215-14-355)
Supplement: Additional file 5 — Work and well-being survey. [file 1745-6215-14-355-S5.docx]

Additional file 5

Work and Well Being Survey

*The following statements are about how you feel at work. Please read each statement carefully and decide if you ever feel this way about your job. If you have never had this feeling, write “0” (zero) in the space preceding the statement. If you have had this feeling, indicate how often you feel it by writing the number (from 1 to 6) that best describes how frequently you feel that way.*

Date **_________** Facility ID **_________** Healthcare Aide ID **__________**

|  | Never | | Almost Never  A few times a year or less | Rarely  Once a month or less | Sometimes  a few times a month | | Often  Once a week | Very Often  a few times a week | Always  Every day | |
| --- | --- | --- | --- | --- | --- | --- | --- | --- | --- | --- |
| 1. At my work I am bursting with energy. | 0 | | 1 | 2 | 3 | | 4 | 5 | 6 | |
| 2.At my job, I feel strong and vigorous | 0 | | 1 | 2 | 3 | | 4 | 5 | 6 | |
| 3. When I get up in the morning, I feel like going to work. | 0 | | 1 | 2 | 3 | | 4 | 5 | 6 | |
| 4. I am enthusiastic about my job. | 0 | | 1 | 2 | 3 | | 4 | 5 | 6 | |
| 5. My job inspires me. | 0 | | 1 | 2 | 3 | | 4 | 5 | 6 | |
| 6. I am proud of the work that I do. | 0 | | 1 | 2 | 3 | | 4 | 5 | 6 | |
| 7. I feel happy when I am working intensely. | 0 | | 1 | 2 | 3 | | 4 | 5 | 6 | |
| 8. I am immersed in my work. | 0 | 1 | | 2 | 3 | 4 | | 5 | | 6 |
| 9. I get carried away when I am working. | 0 | 1 | | 2 | 3 | 4 | | 5 | | 6 |
|  | | | | | | | | | | |
