# Supplementary material for: Sustaining Transfers through Affordable Research Translation (START): study protocol to assess knowledge translation interventions in continuing care settings
Source: Trials. 2013 Oct 26;14:355. doi: 10.1186/1745-6215-14-355 (PMC4231466; doi:10.1186/1745-6215-14-355)
Supplement: Additional file 6 — Questionnaire for licensed practical nurse and facility leader. [file 1745-6215-14-355-S6.doc]

Additional file 6

Questionnaire for Licensed Practical Nurse & Facility Leader

Date **_________** Facility ID **_________**

**Please Check One:** Licensed Practical Nurse__ Facility Leader __

**Perceptions of the Simple Reminders**

1. Do you believe the posters and bedside stickers remind healthcare aide staff to do the sit-to-stand activity? Please explain your answer.

________________________________________________________________

________________________________________________________________

________________________________________________________________

1. Can you think of a better way to remind them to complete the activity?

________________________________________________________________

________________________________________________________________

________________________________________________________________

**Perceptions of the Peer Reminder**

1. Do you think the peer reminder *effectively* *encourages* your health care aide staff to do the sit-to-stand activity with the clients? Please explain your answer.

________________________________________________________________

________________________________________________________________

________________________________________________________________

1. What do you think makes it *easy* for the peer reminder to offer encouragement to the healthcare aide staff completing the sit-to-stand activtiy?

________________________________________________________________

________________________________________________________________

________________________________________________________________

1. What do you think makes it *difficult* for the peer reminder to offer encouragement to the healthcare aide staff completing the sit-to-stand activity?

________________________________________________________________

________________________________________________________________

________________________________________________________________

**Perceptions of the Documentation Flowsheet**

1. What do you think of the flowsheets where staff document the sit-to-stand activity? Are their elements of the flowsheet that make it *difficult* to use? Elements that make the flowsheet *easy* to use?

________________________________________________________________

________________________________________________________________

________________________________________________________________

**Perceptions of the Sit-to-Stand Activity**

1. What do you think of the sit-to-stand activity?

________________________________________________________________

________________________________________________________________

________________________________________________________________
