# Supplementary material for: Sustaining Transfers through Affordable Research Translation (START): study protocol to assess knowledge translation interventions in continuing care settings
Source: Trials. 2013 Oct 26;14:355. doi: 10.1186/1745-6215-14-355 (PMC4231466; doi:10.1186/1745-6215-14-355)
Supplement: Additional file 7 — Interview guide for health care aides. [file 1745-6215-14-355-S7.doc]

Additional file 7

Interview Guide for Healthcare Aides

Date **_________** Facility ID **_________**

**Perceptions of the Paper-based Reminders**

1. Do the posters and beside stickers remind you to do the sit-to-stand activity?
2. Can you think of a better way to remember?

**Perceptions of the Peer Reminder**

1. Has the [name of peer reminder] ever discussed the sit-to-stand activity with you?

If **yes**: What did they say?

1. Does the [name of peer reminder] encourage you to do the sit-to-stand activity with the clients?

If **yes**: How? Can you provide an example?

1. What do you think about the times when the [name of peer reminder] talks to staff about the sit-to-stand activity?

**Perceptions of the Documentation Flowsheet**

1. What do you think of the flowsheet where you write about the sit-to-stand activity?

Prompt: What makes it easy to use? What could make it easier to use?

Prompt: What makes it difficult to use?

**Perceptions of the Sit-to-Stand Activity**

1. How many clients are you taking care of today that are part of the sit-to-stand activity?
2. What do you think of the sit-to-stand activity?

Prompt: Is it helpful for the clients? Is it helpful for you?

Prompt: How important is it for the clients to stay mobile?

Prompt: Is the sit-to-stand activity easy to do? Why / Why not?

Prompt: What other things help the clients to stay mobile?
