# Supplementary material for: Sustaining Transfers through Affordable Research Translation (START): study protocol to assess knowledge translation interventions in continuing care settings
Source: Trials. 2013 Oct 26;14:355. doi: 10.1186/1745-6215-14-355 (PMC4231466; doi:10.1186/1745-6215-14-355)
Supplement: Additional file 8 — Interview guide for peer reminder. [file 1745-6215-14-355-S8.docx]

Additional file 8

Interview Guide for Peer Reminder

Date **_________** Facility ID **_________**

**Perceptions of the Paper-based Reminders**

1. Do the posters and bedside stickers remind the healthcare aides to:

a) do the sit-to-stand activity with the residents? b) complete the documentation flowsheets?

1. Can you think of a better way to help them remember?

**Perceptions of the Peer Reminder Role**

1. How do you encourage healthcare aide staff to:

a) do the sit-to-stand activity with the residents? b) complete the documentation flowsheets?

Prompt: What things do you do or say? Can you provide an example?

1. What response have you received from the healthaide staff when you encourage them to do the sit-to-stand activity?
2. What makes it *easy* to encourage healthcare aide staff to:

a) do the sit-to-stand activity with the clients? b) complete the documentation flowsheets?

Prompt: What kind of support has been helpful?

1. What makes it *hard* to encourage other healthcare aide staff to:

a) do the sit-to-stand activity with the clients? b) complete the documentation flowsheets?

**Perceptions of the Documentation Flowsheet**

1. What do you think of the flowsheet where you write about the sit-to-stand activity?

Prompt: What makes it easy to use? What could make it easier to use?

Prompt: What makes it difficult to use?
